# Stable chalcogenide Ge–Sb–Te heterostructures with minimal Ge segregation

**DOI:** 10.1038/s41598-024-66441-y

**Published:** 2024-07-08

**Authors:** Marco Bertelli, Gianfranco Sfuncia, Sara De Simone, Adriano Diaz Fattorini, Sabrina Calvi, Valentina Mussi, Fabrizio Arciprete, Antonio M. Mio, Raffaella Calarco, Massimo Longo

**Affiliations:** 1 Institute for Microelectronics and Microsystems (IMM), Consiglio Nazionale delle Ricerche (CNR), Via del Fosso del Cavaliere 100, 00133 Rome, Italy; 2 Institute for Microelectronics and Microsystems (IMM), Consiglio Nazionale delle Ricerche (CNR), Strada VIII N. 5, 95121 Catania, Italy; 3 Department of Physics, University of “Tor Vergata”, Via della Ricerca Scientifica 1, 00133 Rome, Italy; 4 Department of Chemical Science and Technologies, University of “Tor Vergata”, Via della Ricerca Scientifica 1, 00133 Rome, Italy

**Keywords:** PCM, Sb_2_Te_3_, GST, Ge, Ge-rich, Sputtering, Crystallization, Ge segregation, Information storage, Condensed-matter physics

## Abstract

Matching of various chalcogenide films shows the advantage of delivering multilayer heterostructures whose physical properties can be tuned with respect to the ones of the constituent single films. In this work, (Ge–Sb–Te)-based heterostructures were deposited by radio frequency sputtering on Si(100) substrates and annealed up to 400 °C. The as-deposited and annealed samples were studied by means of X-ray fluorescence, X-ray diffraction, scanning transmission electron microscopy, electron energy loss spectroscopy and Raman spectroscopy. The heterostructures, combining thermally stable thin layers (i. e. Ge-rich Ge_5.5_Sb_2_Te_5_, Ge) and films exhibiting fast switching dynamics (i. e. Sb_2_Te_3_), show, on the one side, higher crystallization-onset temperatures than the standard Ge_2_Sb_2_Te_5_ alloy and, on the other side, none to minimal Ge-segregation.

## Introduction

Nowadays, the growing demand for embedded electronic devices able to share, process and store huge amounts of data within the Internet of Things (IoT)^1^ is pushing the research institutions and the semiconductor industry to develop new technical solutions to realize cheaper connected electronic devices with both stiff and flexible materials^2–5^. Chalcogenide phase change memory (PCM) alloys are extensively used within the IoT environment to realize non-volatile memories^6^ thanks to their capability to switch, upon the application of electrical stimuli, between two distinct structural solid-states phases (i.e., crystalline and amorphous)^7^. Nevertheless, the use of the standard chalcogenide Ge_2_Sb_2_Te_5_ (GST225) alloy for specific applications is limited because of its low thermal stability. On one side, GST225 layers, which were deposited in our system, show the onset of crystallization at Ton = 140 °C; on the other side, according to JEDEC standard (Global Standards for the Microelectronics Industry^8^) during soldering (temperature peak at 260 °C for 2 min) there is no way to preserve the bit states (i.e. amorphous state which represents the Reset state) and it is a big challenge to fit automotive specifications (ten years at 150 °C). Several solutions have been proposed to increase the thermal stability of Ge–Sb–Te films like the adding of dopants^9^, the choice of suitable capping layers^10^, the modification of crystalline procedures^11^, the incorporation of Se in place of Sb^12^ and the enrichment of the alloy Ge-content^13,14^.

In this work, we investigated a different approach to improve the thermal stability of Ge–Sb–Te layers, namely the deposition of (Ge–Sb–Te) multi-layers (MLs) heterostructures whose physical properties can be tuned with the suitable choice of the constituent chalcogenide films.

We show the results of an experimental study on as-deposited and annealed (Ge–Sb–Te) MLs grown, at room temperature, on Si(100) by radio-frequency (RF)-sputtering. The structural properties of the MLs were studied by means of temperature-dependent X-ray diffraction measurements (XRD) in grazing incidence diffraction (GID), high-angle annular dark field (HAADF), scanning transmission electron microscopy (STEM), electron energy loss spectroscopy (EELS) while the vibrational properties were investigated by means of Raman spectroscopy. The experimental results revealed that, by combining Sb_2_Te_3_, Ge_5.5_Sb_2_Te_5_ and Ge thin films, the obtained heterostructure shows two advantages: (i) a higher crystallization onset temperature (T_on_ = 150/200 °C) than standard GST225 RF-sputtered single layers (T_on_ ~ 140 °C); (ii) minimal Ge-segregation in comparison with Ge-rich Ge_5.5_Sb_2_Te_5_ single layers.

## Results

Different combinations of (Ge–Sb–Te) MLs (Sb_2_Te_3_, Ge, GST225, Ge_5.5_Sb_2_Te_5_) were studied to increase the thermal stability of the grown heterostructures in comparison with the reference alloy GST225, hinder Ge-segregation and get a fast switching dynamics between the amorphous and the crystalline states of the MLs. Therefore, the thermal stability for three pairs of chalcogenide layers was investigated: Sb_2_Te_3_/GST225 (sample A), GST225/Ge (sample B) and Ge_5.5_Sb_2_Te_5_/Ge (sample C). The precise nominal structure of the fabricated MLs is presented in Table 1.

**Table 1:** Scheme of the as-grown (Ge–Sb–Te) (GST)-based heterostructures.

Sample name	Sample structure
A	Si(100)/SiO_2_/[Sb_2_Te_3_ (10 nm)/GST225 (10 nm)]_2_/Sb_2_Te_3_ (10 nm)/Si_3_N_4_(10 nm)
B	Si(100)/SiO_2_/[GST225 (10 nm)/Ge (10 nm)]_2_/GST225 (10 nm)/Si_3_N_4_(10 nm)
C	Si(100)/SiO_2_/[Ge_5.5_Sb_2_Te_5_ (10 nm)/Ge (10 nm)]_2_/Ge_5.5_Sb_2_Te_5_ (10 nm)/Si_3_N_4_(10 nm)
D	Si(100)/SiO_2_/[Sb_2_Te_3_ (10 nm)/GST225 (10 nm)/Ge(10 nm)]_4_/Sb_2_Te_3_ (10 nm)/GST225 (10 nm)/Si_3_N_4_(10 nm)
E	Si(100)/SiO_2_/[Sb_2_Te_3_ (10 nm)/Ge_5.5_Sb_2_Te_5_ (10 nm)/Ge(10 nm)]_2_/Sb_2_Te_3_ (10 nm)/Ge_5.5_Sb_2_Te_5_ (10 nm)/Si_3_N_4_(10 nm)

The composition of reference single layers, which were deposited with the same growth parameters used for the MLs structures, was evaluated with XRF measurements. The XRF data confirmed that, on the one side, the composition of the grown GST and Sb_2_Te_3_ layers well reproduced the nominal composition of the corresponding sputtered targets (Table 2, within 5% error) and, on the other side, the composition of the Ge_5.5_Sb_2_Te_5_ layer was in good agreement with the one which was expected by the co-sputtering of the GST225 and Ge targets.

**Table 2:** XRF measurements on as-grown (Ge–Sb–Te) layers.

Nominal composition	Measured composition		
	Ge (molar fraction)	Sb (molar fraction)	Te (molar fraction)
GST225	2.0	2.0	5.0
Sb_2_Te_3_	–	1.9	3.0
Ge_5.5_Sb_2_Te_5_	5.4	1.9	5.0

**Figure 1:**
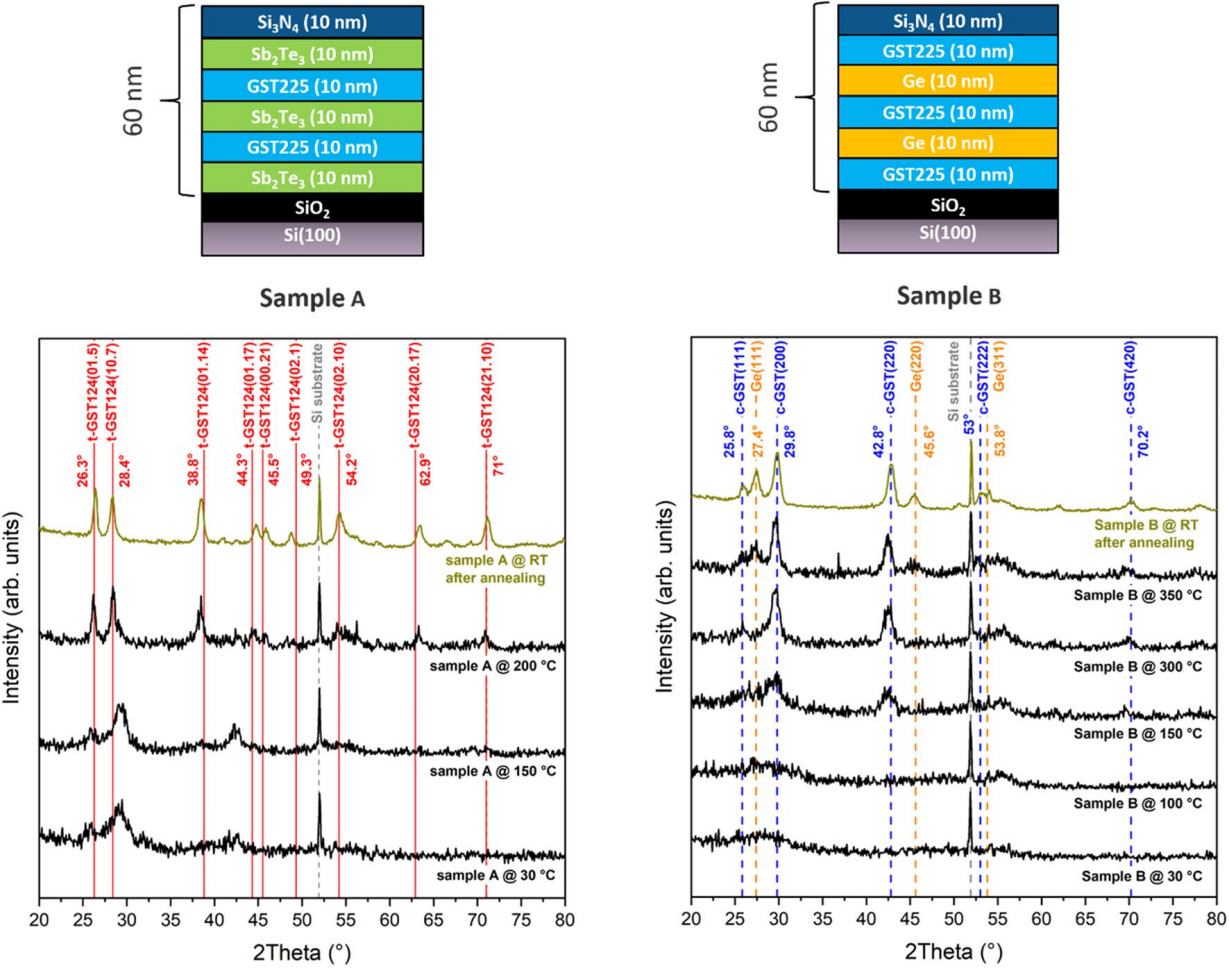
X-ray GID (ω − 2θ) scans on (bottom) sample A and (top) sample B. The scans during annealing (T = 30/400 °C, black color) and after annealing (RT, brown yellow color) were acquired for 25’ and 3 h, respectively. The dotted lines indicate the peak positions for the experimental GID (ω − 2θ) scan of c-GST225 (blue color) and Ge (orange color); the continuous red lines indicate the peak positions for simulated GID (ω − 2θ) scan of t-GST124.

Figure 1 (left) shows the GID X-ray (ω − 2θ) scans of the sample A during annealing in N_2_ atmosphere (T = 30/400 °C). The GID measurement at 30 °C shows that the as-grown heterostructure Sb_2_Te_3_/GST225 is not completely amorphous: the broad peaks appearing at ~ 25.8°, 29.4° and 42.5° could be attributed to crystalline Sb_2_Te_3_. The sample crystallization further develops with temperature increasing from 150 to 200 °C: the GID scan at 200 °C shows three peaks at 26.3°, 28.4° and 38.8°. The spectrum of quenched crystalline sample A, after annealing at 400 °C, does not show typical reflexes of crystalline Sb_2_Te_3_ and/or GST225. The assignment of the peaks was done by comparison of the experimental data with the simulated VESTA XRD spectrum of trigonal (t-)Ge_1_Sb_2_Te_4_^15,16^. Such an assignment is supported by two observations: on one side, it has already been observed that GeTe and Sb_2_Te_3_ superlattices, after annealing at 400 °C, intermix at the interfaces by generating the t-Ge_1_Sb_2_Te_4_ alloy^17^; on the other side, the calculated medium composition of sample A in the hypothesis of complete intermixing (Ge_0.06_Sb_0.35_Te_0.59_) is quite close to the composition of Ge_1_Sb_2_Te_4_ (Ge_0.14_Sb_0.29_Te_0.57_). According to peak attribution of the GID scan of sample A (Fig. 1 (left)) to the t-Ge_1_Sb_2_Te_4_ compound, the peaks at 26.3°, 28.4°, 38.8°, 44.3°, 45.5°, 49.3°, 54.2°, 62.9° and 71° can be assigned to the (01.5), (01.7), (01.14), (01.17), (01.21), (02.1), (02.10), (20.17) and (21.10) reflexes of the t-Ge_1_Sb_2_Te_4_ alloy, respectively.

Figure 1 (right) shows the GID X-ray (ω − 2θ) scans of the sample B during annealing in N_2_ atmosphere (T = 30/400 °C). The GID measurement at 30 °C shows that the as-grown GST/Ge heterostructure is amorphous. The onset of crystallization of the sample takes place by increasing the T from 100 to 150 °C: the peaks at 29.8° and 42.8° can be assigned, by comparison with the GID spectra of crystalline cubic GST (c-GST), to the c-GST(200) and c-GST(220) reflection, respectively. By further increasing the T to 400 °C, the formation of Ge crystalline grains and additional c-GST is observed: the peaks at 25.8°, 27.4° and 45.6° are assigned to the crystalline reflections c-GST(111), c-Ge(111) and c-Ge(220), respectively.

The GID X-ray (ω − 2θ) scans of the sample C (not shown) show that the onset of crystallization of the amorphous as-grown heterostructure takes place with T increasing from 200 to 250 °C. By increasing the T to 400 °C, the formation of crystalline c-GST (150 °C < T < 200 °C) and Ge (300 °C < T < 350 °C) phases is observed.

In order to increase, in comparison with GST225 as well as sample A and B, the onset crystallization temperature and reduce the Ge-segregation, which is typical of Ge-rich layers (sample C), two chalcogenide heterostructures (sample D and E) were grown, annealed and investigated with GID, STEM, EELS and Raman spectroscopy. Sample D combines the structure of both sample A and sample B and was investigated to see if T_on_ could be increased by introducing Ge-layers between the Sb_2_Te_3_/GST225 bi-layers. Sample E is similar to sample D, but with the replacement of GST225 with a Ge-rich GST layer to investigate if the Ge layer might help in the mitigation of the Ge segregation which was observed for heterostructures with Ge-rich GST/Sb_2_Te_3_ bi-layers (sample C).

**Figure 2:**
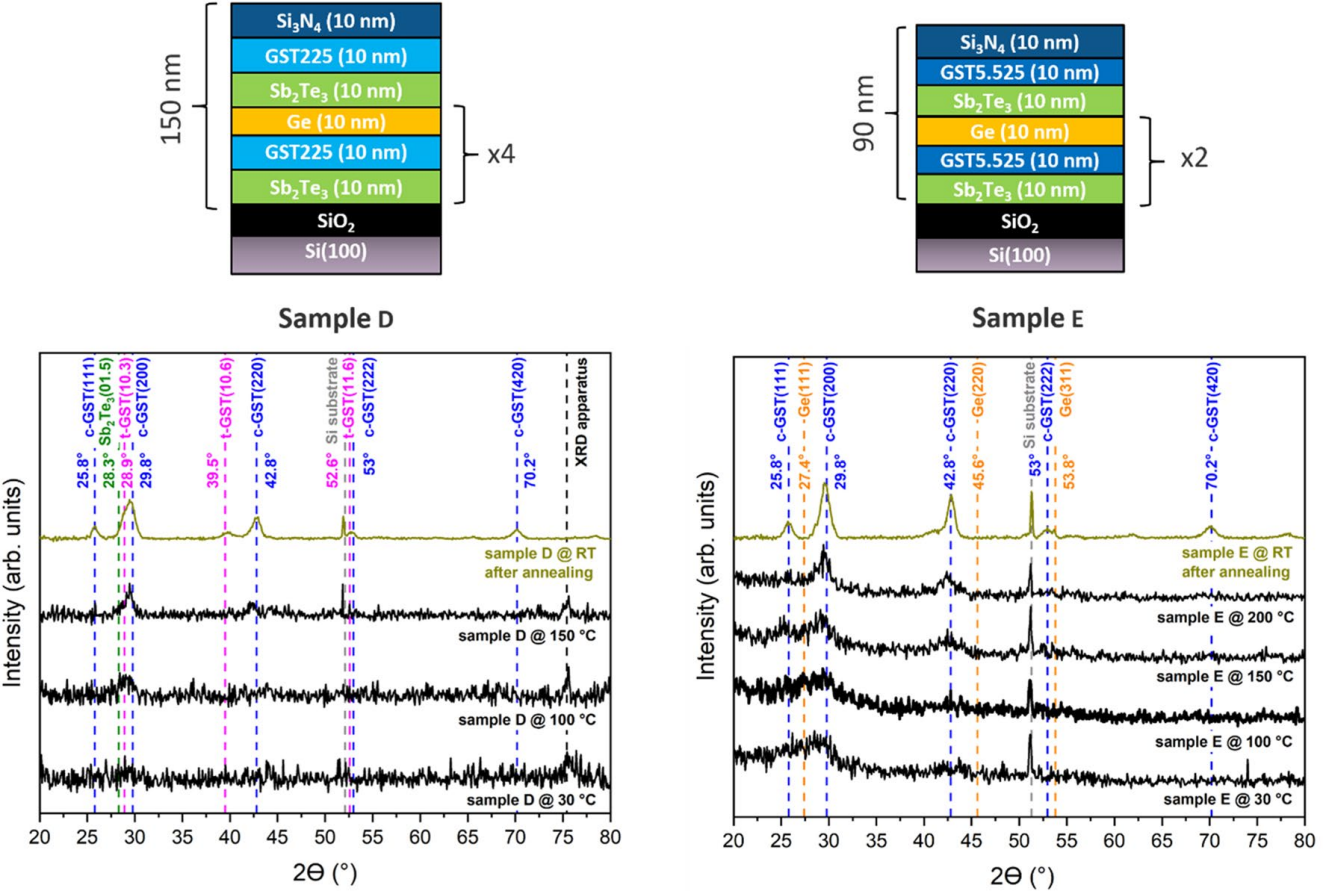
GID (ω − 2θ) scans on (left) sample D and (right) sample E. The scans during annealing (T = 30/400 °C, black color) and after annealing (RT, brown yellow color) were acquired for 25’ and 3 h, respectively. Dotted lines indicate the positions of the GID (ω − 2θ) scan peaks for crystalline Sb_2_Te_3_ (green color), c-GST225 (blue color), t-GST225 (purple color) and Ge (orange color).

Figure 2 (left) shows the GID X-ray (ω—2θ) scans of the sample D during annealing in N_2_ atmosphere (T = 30 ÷ 400 °C).

The GID measurement at 30 °C shows that the as-grown heterostructure is amorphous. The onset of crystallization takes place by increasing the T from 100 to 150 °C: the peak at 29.8° and 42.8° can be assigned, by comparison with the GID spectra of crystalline GST225, to the c-GST225(200) and c-GST225(220) reflections, respectively. By further increasing the temperature to 400 °C, the formation of other crystalline c-GST225 orientations is observed. After sample quenching, the presence of crystalline grains of Sb_2_Te_3_ and t-GST cannot be excluded: the peaks at 28.3°, 28.9° and 39.5° could be assigned to the Sb_2_Te_3_(01.5), t-GST225(10.3) and t-GST225(10.6) reflections, respectively. The comparison of the XRD spectra for sample D (Fig. 2, left) with the ones for sample A (Fig. 1, left) shows that, by adding a Ge-layer to each GST225/Sb_2_Te_3_ bilayer, the onset temperature of crystallization does not increase (T_on_ = 100/150 °C), while the intermixing between the GST225 and Sb_2_Te_3_ layers does not evolve in the formation of t-Ge_1_Sb_2_Te_4_ crystals.

**Figure 3:**
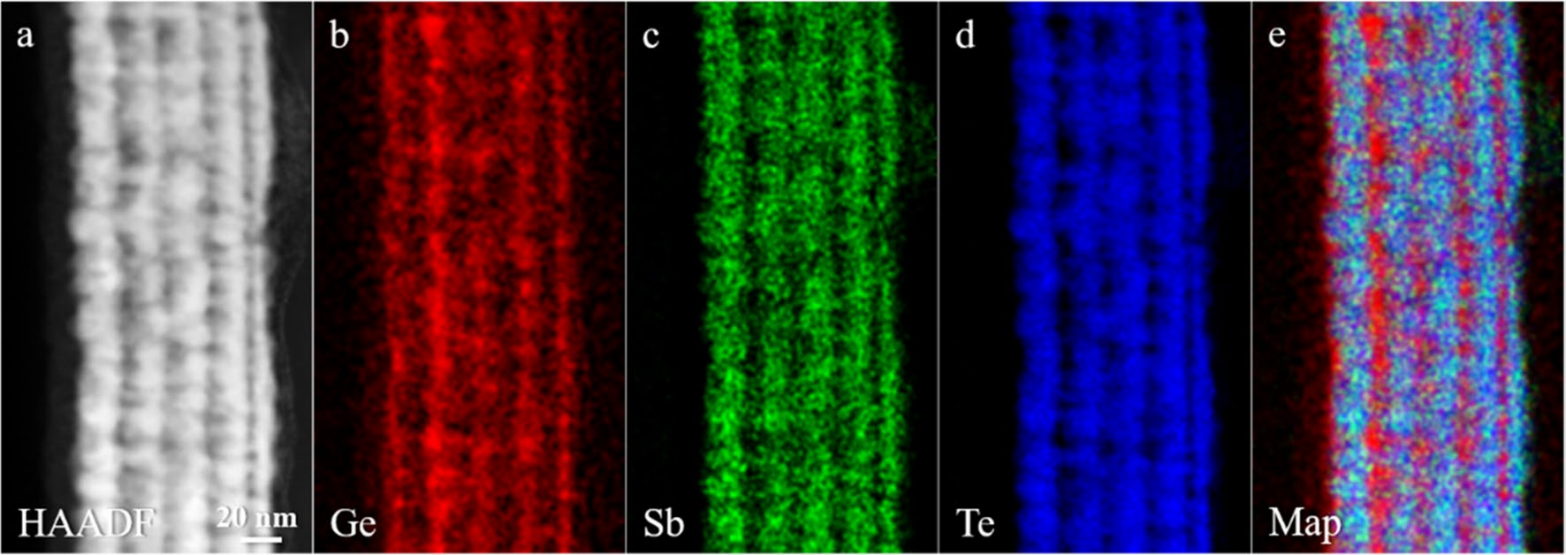
(**a**) HAADF STEM image of the ML heterostructure of sample D after annealing at 400 °C (substrate on the left, capping layer on the right of the ML heterostructure). (**b**) Ge EELS map obtained from Ge L-edge at 1217 eV. (**c)** Sb EELS map obtained from Sb M-edge at 528 eV. (**d**) Te EELS map obtained from Te M-edge at 572 eV. (**e**) Composite map of Ge, Sb and Te EELS maps.

Figure 3a shows a HAADF STEM image of the MLs heterostructure of sample D after annealing at 400 °C. The intensity of HAADF STEM signal is roughly proportional to *Z*^1.7^, being *Z* the atomic number of the elements probed by the electron beam (*Z*-contrast). For this reason, it is possible to associate brighter and darker regions of the heterostructure to the heavier (GST225/Sb_2_Te_3_) and lighter (Ge) layers, respectively. Sample D was further investigated by combining STEM with EELS in Spectrum Imaging (SI) mode, to obtain space-resolved distributions of Ge, Sb and Te in the MLs heterostructure. Figure 3b–d report the chemical maps of Ge, Sb and Te obtained from EELS signals (L-edge at 1217 eV for Ge; M-edge at 528 eV and 572 eV for Sb and Te, respectively). Figure 3e shows a composite map of Ge, Sb and Te, evidencing the presence of Ge layers and the overlap of the Sb and Te signals, corresponding to the original GST225 and Sb_2_Te_3_ layers. While the locations of the Ge-rich layers are well visibile, Ge diffuses also in the Sb_2_Te_3_ layers nearby.

Figure 2 (right) shows the GID X-ray (ω − 2θ) scans of sample E during annealing in N_2_ atmosphere (T = 30/400 °C). In particular, the GID measurement at 30 °C shows that the as-grown heterostructure is almost entirely amorphous. The broad peak at ~ 28.3° could be attributed to the (01.5) reflection of crystalline Sb_2_Te_3_. The onset of crystallization of the sample takes place by increasing the temperature from 150 to 200 °C: the peaks at 29.8° and 42.8° can be assigned, by comparison with the GID spectra of crystalline c-GST225, to the c-GST225(200) and c-GST225(220) reflections, respectively. By further increasing the temperature to 400 °C, the formation of other crystalline c-GST225 grains is observed: the peaks at 25.8° and 70.2° can be assigned to the c-GST225(111) and c-GST225(420) reflections, respectively. The comparison of the XRD spectra for sample E (Fig. 2—right) with those for sample D (Fig. 2—left), shows that, by substituting the GST225 layer with the Ge-rich Ge_5.5_Sb_2_Te_5_ layer, the onset temperature of crystallization increases (T_on_ = 150/200 °C) and, at the same time, none to minimal Ge-segregation is observed.

**Figure 4:**
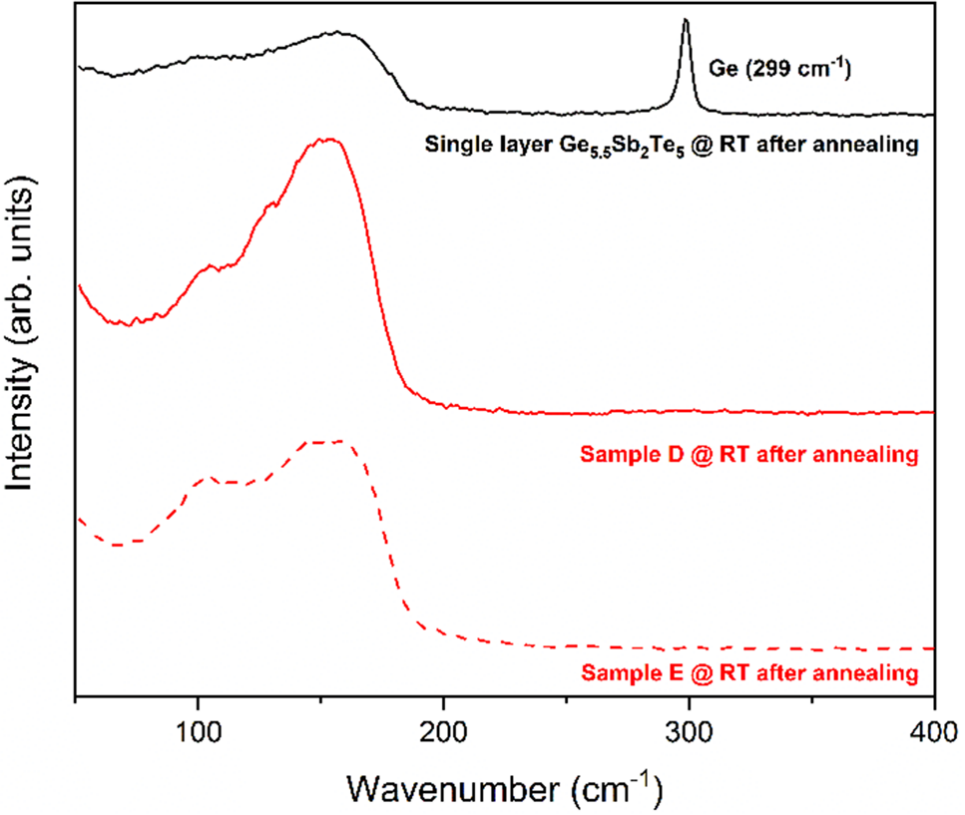
Raman spectra of sample D, E and single layer Ge_5.5_Sb_2_Te_5_ @ RT after annealing at 400 °C.

In case the XRD scans of the annealed samples did not show any peak which could be attributed to Ge, Raman measurements were performed to assess the possible formation of tiny Ge nanocrystals (grain-size smaller than few nm). Figure 4 shows the Raman spectrum acquired at RT of the annealed (T = 400 °C) samples D and E, compared with the Raman spectrum of an annealed single Ge_5.5_Sb_2_Te_5_ layer (thickness = 185 nm). The Raman data on sample E do not show the presence of crystalline Ge-grains in the annealed MLs structure: the typical feature at 299 cm^−1^, which in the literature is assigned to crystalline Ge^18–20^, is not visible. In both Raman spectra of sample E and single Ge-rich Ge_5.5_Sb_2_Te_5_ layer, the band feature at frequencies lower than 190 cm^−1^ covers the frequency range in which characteristic Ge-Te and Sb–Te vibrational modes of c-GST225 are located. The peaks at 105 cm^−1^ and 165 cm^−1^ in the Raman spectrum of c-GST225 layers, grown by pulsed laser deposition, have been assigned to the A_1_ vibrational mode of corner sharing tetrahedra GeTe_4_ and the A_1g_ vibrational mode of Sb_2_Te_3_ units, respectively^21,22^.

**Figure 5:**
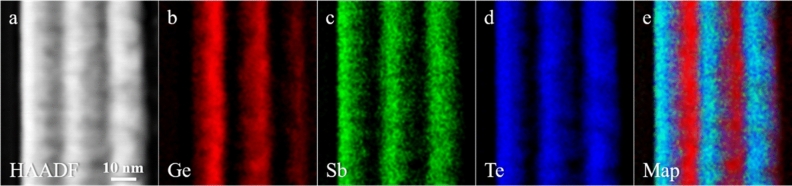
(**a**) HAADF STEM image of the ML heterostructure forming sample E, as-grown (substrate on the left, capping layer on the right of the ML heterostructure). (**b**) Ge EELS map obtained from the Ge L-edge at 1217 eV. (**c**) Sb EELS map obtained from the Sb M-edge at 528 eV. (**d**) Te EELS map obtained from the Te M-edge at 572 eV. (**e**) Composite map of the Ge, Sb and Te EELS maps.

Figure 5a shows a HAADF STEM image of the MLs heterostructure of sample E, as-grown. The chemical maps of Ge, Sb, Te, as well as the composite, are reported in Fig. 5b–e, respectively. The scope of such investigation was to rule out the Ge segregation prior to crystallization. Indeed, the GID spectrum of the as-grown sample exhibited a broad diffraction signal and Ge segregation had previously been detected in an annealed single-layer Ge_5.5_Sb_2_Te_5_ sample (not shown). Indeed, no evidence of Ge segregation has been observed in Ge_5.5_Sb_2_Te_5_ layers embedded in the heterostructures of sample E. Compared to Fig. 3, in this as-grown sample E, the chemical maps of Ge, Sb and Te indicate lower diffusion of Ge into the adjacent Sb_2_Te_3_ layers.

## Discussion

Overall, the scope of the present work was to demonstrate that the Ge segregation might be limited by the small layer volume of the Ge-rich alloy inserted in a ML structure, maintaining a reasonably high crystallization temperature. For better comparison the results are summarized in Table 3.

**Table 3:** Scheme of the as-grown (Ge–Sb–Te) (GST)-based heterostructures with their T_on_ and Ge segregation evaluation from the GID results.

Sample name	Schematic sample structure	T_on_ (°C)	Ge segregation
A	Sb_2_Te_3_/GST225	150–200	No
B	GST225/Ge	100–150	Yes
C	Ge_5.5_Sb_2_Te_5_/Ge	200–250	Yes
D	Sb_2_Te_3_/GST225/Ge	100–150	No
E	Sb_2_Te_3_/Ge_5.5_Sb_2_Te_5_/Ge	150–200	No

At first, from Table 3 it can be observed that the T_on_ of sample A is higher than what expected for GST225; such a surprising result can be explained by the fact that the layers elements intermixed to form GST124 and crystallized directly in a stable trigonal form. Interestingly, sample D, that has the same structure of sample A, but with the presence of Ge layers, does not show the formation of t-GST124, meaning that the Ge layers hinder the full intermixing of GST225 and that Sb_2_Te_3_ is maintained upon annealing. However, the Ge layer might also partially intermix with the other layers. Considering the value of T_on_ shown by sample B, containing GST225 and Ge, we infer that Ge dissolved into the GST225 layer to form an alloy with at least 40% of Ge^23^; such an amount was shown to be the minimum Ge content necessary for the formation of segregated Ge^24^. The Ge layers were partially preserved after crystallization, as clearly shown by the EELS measurements of sample D. In the case of amorphous pure Ge layers grown with our sputtering system, the onset of Ge-crystallization was observed, during samples annealing, with T no higher than 500 °C. By comparison with the experimental results from sample B, it is reasonable to presume that the interfaces between GST225 and Ge act as nucleation centers and promote the crystallization of Ge. The enhanced crystal growth, upon temperature increase, due to the presence of interfaces between chalcogenide thin films, has already been observed for GeTe/Sb_2_Te_3_ superlattices grown by physical vapour deposition^25^ and GST225/Sb_2_Te_3_ heterostructures grown by pulsed laser deposition^26^.

The XRD results of sample C confirmed this intermixing mechanism, as well. Nevertheless, the presence of crystalline Ge in the quenched sample C might be due not only to the presence of interfaces between the Ge_5.5_Sb_2_Te_5_ and Ge layers, but also to the segregation of Ge from the Ge-rich Ge_5.5_Sb_2_Te_5_ layers, as already observed for annealed Ge-rich Ge_x_Sb_y_Te_z_ films grown by sputtering^20^, molecular beam epitaxy^27^ and physical vapour deposition^13^.

Importantly, sample E revealed that heterostructures containing Ge-rich GST, Sb_2_Te_3_ and Ge layers showed a higher crystallization temperature than GST225 layers and minimal Ge segregation.

In conclusion, (Ge–Sb–Te) MLs heterostructures were grown by means of RF-sputtering, at room temperature, on Si(100) substrates and were investigated by XRD, STEM, EELS and Raman spectroscopy. The MLs which combine thermally stable thin layers (i.e. Ge_5.5_Sb_2_Te_5_ and Ge) with fast switching layers (i.e. Sb_2_Te_3_) show, on the one side, higher onset crystallization temperature (T_on_ = 150–200 °C) than the standard GST225 alloy (T_on_ = 140 °C) and, on the other side, none to minimal Ge-segregation, as opposed to Ge-rich Ge_5.5_Sb_2_Te_5_ single layers. The obtained results are a first step towards the fabrication/characterization of thermally stable PCM-cells based on Ge_5.5_Sb_2_Te_5_/Sb_2_Te_3_/Ge MLs, at the same time exhibiting lower Ge-segregation than PCM cells based on single layers of Ge-rich Ge_x_Sb_2_Te_5_^28^, for future electrical tests.

## Methods

### Samples growth

The (Ge–Sb–Te) MLs heterostructures were deposited by RF-sputtering in a custom-made high vacuum chamber system (IONVAC PROCESS srl, Pomezia, Italy), equipped with a planetary system for deposition and four confocal targets. The heterostructures were grown at room temperature (RT) on Si(100) substrates (p-type, ρ = 1/10 Ω cm) with a thin native oxide layer on top. The GST225, Sb_2_Te_3_ and Ge targets were provided by Robeko GmbH & Co. KG (Mehlingen, Germany), with a 99.99%, 99.99% and 99.999% purity, respectively. The chamber pressure was in the range of low 10^–2^ mbar.

### X-ray fluorescence (XRF)

Energy dispersive X-ray Fluorescence (XRF) measurements were performed *ex-situ* using a RIGAKU Nex DE VS spectrometer (Applied Rigaku Technologies Inc., Austin, Texas, USA) equipped with a 60 kV X-ray tube and a silicon drift detector.

### X-ray diffraction (XRD)

XRD measurements were performed ex-situ by a D8 Discover diffractometer (BRUKER, Billerica, Massachusetts, USA) equipped with a Cu X-ray source (Cu-Kα_1_ radiation λ = 1.54 Å, 40 kV and 40 mA) and a DHS1100 dome-type heating stage (ANTON PAAR, Graz, Austria) for temperature measurements in N_2_ atmosphere. Grazing incidence diffraction (GID) (ω − 2θ) scans were acquired during annealing with the following parameters:

- Temperature steps at T = 30, 100, 150, 200, 250, 300, 350 and 400 °C.
- Temperature gradient = 60 °C/min.
- Step acquisition time = 25’.

Finally, the X-ray GID scans after annealing were acquired at RT for 3 h.

### Raman

Raman spectra were acquired ex situ by means of a DXR2xi Raman imaging microscope (Thermofischer, Waltham, Massachusetts, USA) equipped with a 532 nm laser source and a 50× objective. The Raman data acquisition was performed at RT in back-scattering geometry, by using a 4mW laser power at the sample surface.

### Scanning transmission electron microscopy and electron energy loss spectroscopy

The local structure, morphology and the element distribution were investigated by scanning transmission electron microscopy (STEM) and electron energy loss spectroscopy (EELS). The analyses were performed by using a JEOL ARM200F Cs-corrected microscope, equipped with a cold-field emission gun and operating at 200 keV. Micrographs were acquired in Z-contrast mode by high-angle annular dark field (HAADF). A GIF Quantum ER system was used for EELS measurements. Both low and core-loss EELS spectra were acquired with the Dual EELS tool, in spectrum imaging (SI) mode.

## Data Availability

The data that support the findings of this study are available from the corresponding author upon reasonable request.
